# Development and validation of an individual-based state-transition model for the prediction of frailty and frailty-related events

**DOI:** 10.1371/journal.pone.0290567

**Published:** 2023-08-24

**Authors:** Aubyn Pincombe, Hossein Haji Ali Afzali, Renuka Visvanathan, Jonathan Karnon

**Affiliations:** 1 College of Medicine and Public Health, Flinders University, Bedford Park, SA, Australia; 2 Aged & Extended Care Services (Geriatric Medicine), Acute and Urgent Care, The Queen Elizabeth Hospital, Central Adelaide Local Health Network, SA Health, Woodville South, SA, Australia; Instituto Nacional de Geriatria, MEXICO

## Abstract

Frailty is a biological syndrome that is associated with increased risks of morbidity and mortality. To assess the value of interventions to prevent or manage frailty, all important impacts on costs and outcomes should be estimated. The aim of this study is to describe the development and validation of an individual-based state transition model that predicts the incidence and progression of frailty and frailty-related events over the remaining lifetime of older Australians. An individual-based state transition simulation model comprising integrated sub models that represent the occurrence of seven events (mortality, hip fracture, falls, admission to hospital, delirium, physical disability, and transitioning to residential care) was developed. The initial parameterisation used data from the Survey of Health, Ageing, and Retirement in Europe (SHARE). The model was then calibrated for an Australian population using data from the Household, Income and Labour Dynamics in Australia (*HILDA*) Survey. The simulation model established internal validity with respect to predicting outcomes at 24 months for the SHARE population. Calibration was required to predict longer terms outcomes at 48 months in the SHARE and HILDA data. Using probabilistic calibration methods, over 1,000 sampled sets of input parameter met the convergence criteria across six external calibration targets. The developed model provides a tool for predicting frailty and frailty-related events in a representative community dwelling Australian population aged over 65 years and provides the basis for economic evaluation of frailty-focussed interventions. Calibration to outcomes observed over an extended time horizon would improve model validity.

## Introduction

Frailty is a common progressive condition among older people (aged 65 or more) with a significant impact on demand for healthcare services. With an ageing population, frailty is becoming a worldwide health concern [1–4]. There are two broad approaches used to measure frailty status: a Frailty Phenotype (FP) and a Frailty Index (FI) [5]. The FI approach measures frailty as a cumulation of deficits across a large range of tests, whereas Frailty Phenotype conceptualises frailty as physiologic syndrome which is present when three or more of five deficits are present [6]. The five deficits are Weight loss, Exhaustion, Low physical activity, Grip strength and Slowness. A score of 0 is classified non-frail, a score of 1 or 2 is classified as pre-frail and a score of 3 or greater is considered frail. Pre-frailty also increases risk of adverse outcomes [6]. Globally, it has been estimated that between 4% and 17% of older people are frail [6]. A recent geospatial modelling study in Australia found that in 2016 over 11% (415,000) of the 3.7 million older people were frail with an additional 46% (1.7 million) at increased risk of becoming frail (pre-frail), representing an increase of 20.5% in the combined frail and pre-frail populations in Australia since 2011 [7]. Frailty confers an increased lifetime risk of important health outcomes/events such as hip fracture, physical disability, and delirium resulting in an increased use of healthcare resources including hospital admissions, doctor consultations, and pharmaceuticals [6, 8–11]. A recent systematic review has shown that frailty is a strong predictor of nursing home placement [12]. These consequences are significant from the perspective of frail people, their families/carers, health and aged care systems.

There is evidence that frailty is potentially manageable and that timely interventions can reduce the risk of adverse events, delay frailty progression and improve frailty status [13–16]. A range of interventions, such as physiotherapy/exercise have been shown to be effective in managing frailty in older people [15, 17–19]. However, the associated costs of these interventions must be assessed to generate cost-effectiveness evidence as one of the key inputs required to inform public funding decisions in countries such as Australia and the UK. A few within-trial economic evaluations have failed to establish cost-effectiveness of frailty interventions [20, 21], however, the time horizon of clinical trials may not be sufficient to capture all important costs and health outcomes of interventions.

The long-term analysis of costs and health outcomes associated with healthcare interventions to assess their value is achieved using decision analytic models. While there are models for predicting frailty and particular events such as mortality [22], using alternate measure for frailty [23], to our knowledge, to date there is only one decision analytic model that can be used to evaluate both costs and outcomes of frailty interventions beyond the duration of clinical studies [24]. Karnon et al. developed a cohort-based state transition (Markov) model to predict costs and quality-adjusted life years (QALYs) over the lifetime of a frail population. The model extrapolated the findings from a clinical trial of a physiotherapy-based intervention from a one-year to a lifetime horizon. Over a 12-month follow-up period, the clinical trial reported a statistically significant reduction in frailty prevalence in the intervention group, a difference in mean costs per patient of $2,145 between the control and intervention groups, but only a trivial difference in quality of life [21]. Using a Markov model to extrapolate costs and outcomes over a lifetime horizon, Karnon et al. estimated an incremental cost per additional QALY of $8,179.

The existing cohort-based Markov model for frailty captured only a limited range of health states, combining frailty-related mediators and outcomes; falls, fractures, transition to residential care and depression [24]. Subsequent research to define an optimal model structure for a frailty cost-effectiveness model found a more complex model structure is required to more accurately capture important differences in costs and outcomes [25]. Given the complexity of the developed model structure and the number of health states required, it is proposed that an individual-based model is preferred [26]. Such models are the preferred modelling choice when there is baseline heterogeneity in the disease being modelled, there are continuous disease markers, time-varying event markers, and prior events have an influence on subsequent event rates [27, 28].

In this paper, we report on the development and validation of an individual-based state transition model that predicts the progression of frailty and frailty-related events over the remaining lifetime of older Australians. The model forms the basis for a cost-effectiveness model for the economic evaluation of interventions to prevent or better manage frailty in community dwelling older people in Australia.

## Methods

### Model overview

A comprehensive model conceptualisation process was undertaken by Afzali et al. [25] to propose a model structure for the cost-effectiveness analysis of frailty interventions. This process was informed by two steps, which involved a critical analysis of frailty-related clinical and economic literature followed by a Delphi study involving clinical experts to achieve consensus on the final proposed model structure. The study identified health states and events for which frailty is a strong independent risk factor as well as patient attributes which influence disease progression. The seven health states/events identified in the final round were hip fracture, falls, transition to residential care, hospital admission, physical disability, delirium, and death. The attributes identified were age, gender, education, frailty status, diabetes, depression, polypharmacy and stroke.

**Fig 1:**
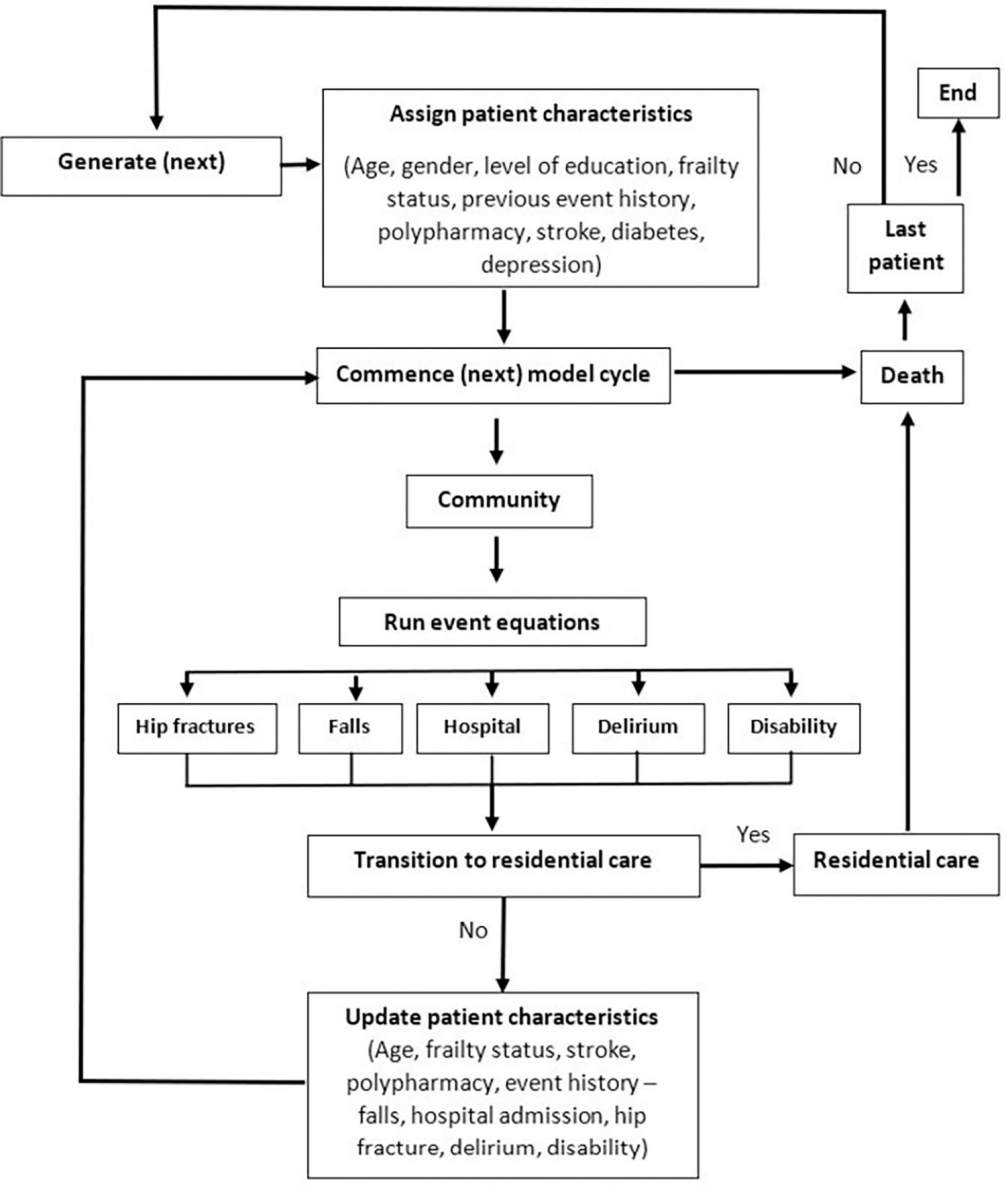
Model structure. In the simulation model, the experience of a new stroke was assumed to impact the likelihood of future events for the remainder of participants’ time horizon (i.e., the participant’s attribute for stroke is permanently set to a value of 1). After experiencing any of the other events represented in the model, the corresponding attributes are set to a value of 1 for 24 cycles, after which, should there be no new event, the value reverts to zero. For participants entering the model (in cycle 0) with an event attribute set to a value of 1 (other than for stroke), the attribute reverts to a value of 0 after 12 months, unless a new event occurs in that time. A participant’s frailty status is updated at the commencement of each cycle. Participants leave the model at age 100 years or when they die.

The flow of individuals through the simulation model is demonstrated in Fig 1. Individuals enter the model with a set of patient attributes, including a frailty phenotype score, and previous event history. These characteristics influence an individual’s predicted risk of outcomes in each monthly cycle. Individuals may experience several events or none in any individual cycle. Patients’ individual characteristics, including frailty status, and event history are then updated prior to beginning the next cycle. A monthly cycle was chosen since it was considered sufficient to provide a reasonable time horizon in relation to disease progression.

### Model construction

The model was constructed using TreeAge software [29]. The model draws participants from a table of individuals with baseline characteristics. These characteristics are then used to predict movement through a combination of event pathways using risk equations at each branch. The model uses Monte Carlo simulations at each event node to reflect stochastic variability in patient trajectories.

The Monte Carlo simulation approach uses tracker variables to update the participants characteristics over time. Events are recorded at each branch by trackers which allow these events to be collected over the course of the model. Updates to patient characteristics and event history are also enabled using trackers and all of these are updated at the end of each cycle.

### Model inputs

#### Data sources

Survey data was used because this is the only data available which captures all the events included in the model structure and allows the creation of a measure of frailty phenotype, a measure which has been used extensively in the frailty literature [24, 30–33]. The review undertaken by Afzali et al. [25] identified The Survey of Health, Ageing and Retirement in Europe (SHARE) as a relevant database to populate the model [34]. The SHARE database includes information on relevant health states, sociodemographic data, and relevant attributes [35]. SHARE comprises longitudinal cross-national data from approximately 140,000 individuals aged 50 years and older covering 28 European countries and Israel. To date, SHARE has collected and released eight panel waves (2004–2021), with participant interviews conducted approximately every two years. To inform the transition probabilities for events and stroke, polypharmacy and frailty status, we used data from waves 4 to 6 (2013 to 2017) [36–43]. This comprised a population of around 26,000 initial participants.

To construct our risk model for residential care, we also used data available from the Australian Institute of Health and Welfare (AIHW) GEN Aged Care Data [44, 45] and the Australian Bureau of Statistics (ABS) [46] to estimate the proportions of different aged cohorts moving into residential care.

To conduct the external validation of the model we used waves 13 and 17 (years 2013 and 2017) of the Household, Income and Labour Dynamics in Australia (HILDA) Survey [47, 48]. HILDA is a household-based panel study, which collects information on economic and personal well-being. Started in 2001, HILDA has released 20 annual waves to date. A health-specific questionnaire module has been run every 4 years beginning from wave 9. Using the HILDA dataset, we were provided with health data for around 2,700 participants who had completed both the wave 13 and the wave 17 surveys for the Health Module [49]. These waves were chosen to their proximity in time frame to the SHARE data waves which had been used to populate the model.

A detailed description of all the variables used to populate the model and to construct the four-factor modified Frailty phenotype in the SHARE and HILDA data are provided in the Appendices.

#### Data analysis

In our model, we used a four-level frailty status variable, representing 0, 1, 2 and 3 or more frailty phenotypes. Individuals with three to five phenotypes were combined into a single category due to the small numbers of observations in the SHARE data.

Derivation of risk equations: Transition probabilities for all events except death and residential care were estimated using multivariable logistic equations in which the dependent variables were the wave 5 recorded values for the event and the independent variables were selected from wave 4 characteristics and events, including the dependent variable event for wave 4. We used a stepwise approach through backwards elimination, beginning with a model which includes all predictors and previous events. Variables were retained in the models if the p value associated with the estimated coefficient was < 0.05. Probability of death was similarly estimated from a logistic regression model in which the dependent variable was death within 12 months.

Due to the very low experience of residential care in the SHARE data, we used a logistic model predicting the probability of transitioning to residential care as a function of age and frailty phenotype only using the SHARE data in wave 4 and 5 [24]. Odds ratios representing the likelihood of pre-frail and frail individuals transitioning to residential care compared to non-frail individuals were converted to relative risks, which were applied to annual probabilities of non-frail individuals transitioning to residential care for 5-year age groups. The probabilities for non-frail individuals transitioning were calibrated such that the aggregate numbers of non-frail, pre-frail and frail individuals transitioning to residential care equalled the observed numbers transitioning to residential care in each age group.

Frailty status was updated each cycle based on a multinomial logistic regression model, which predicted the probability of moving from one frailty phenotype level to another based on individuals’ current frailty phenotype level, attributes and prior events. In the frailty status prediction model, age was entered as a factor variable in 5 year age bands, from ages 65 to 69 years, with the last category being age 85 years and above. Age was entered as a continuous variable for all other sub-models.

All analyses were performed using Stata v14.

All the individual model specifications are provided in the Appendices.

### Model performance evaluation

The face validity of the model structure and the choice of data sources was confirmed by experts through the process described in Afzali et al. [25]. Verification of the model involved a process of checking all formulae, model debugging and checking that the outputs were logical. Internal validation was performed by comparing the overlap in the 95% and 99% confidence intervals around the observed and predicted means for event incidence for individuals at 24 months and at 48 months with the observed mean and confidence intervals for the incidence of events in the SHARE wave 5 and wave 6 data for the wave 4 participants. We also checked whether the estimated mean incidence fell within 10% of the mean observed value for each event. We compared the model predictions for the rates of polypharmacy and the mean frailty phenotype score at 24 months and at 48 months using the same criteria. Due to growing evidence of gender differences in the progression of frailty and frailty characteristics [30, 50–52], we compared model predictions for individuals 65–75 years of age and for ages over 75 years separately for men and women.

We assessed the external validity of the model by using waves 13 and 17 of the HILDA data [47]. Individual participant baseline values for falls, hip fracture and delirium, for which there were no equivalent values in the HILDA data, were imputed using logistic associative models in the SHARE wave 4 data which predicted the probability of an individual reporting a history of these characteristics based on other variables in the wave 4 data. Using a random number generator to add stochastic variability, these predicted probabilities were then used to assign baseline values for these variables for each individual in the HILDA data.

We then compared model predicted outcomes to observed outcomes (excluding falls, hip fracture and delirium) in the HILDA data over 48 months for participants who completed both surveys 13 and 17. Pre-specified convergence criteria for the external validation targets required prediction of the events and attributes at 48 months to within the 95% confidence interval of the observed values in each subgroup for age and gender.

#### Model calibration

As our model did not meet all of the pre-specified convergence criteria for internal and external validation, model calibration was undertaken. To calibrate the simulation model to the HILDA data, we modified a method used by Kim and Thompson (2010), which involved the application of multipliers to selected coefficients in the predictive equations [53]. Due to the number of models and the number of variables in our models, we modified Kim and Thompson’s approach to apply the multipliers directly to the transition probabilities, e.g., [multiplier for probability of death x model predicted probability of Death].

To estimate multipliers for each prediction model, we first used multinomial functions to randomly sample 2,000 sets of coefficient values across all prediction sub models using the coefficient and variance-covariance matrices for the originally specified model equations. The multipliers were adjusted incrementally until the mean outputs across the 2,000 model runs using the sampled sets of coefficient values met the convergence criteria for all external validation targets.

We then ran 2,000 model runs using the calibrated multipliers to identify sets of coefficient values that individually met the convergence criteria. The final model was then run again using the convergent parameter sets.

## Results (Model performance)

The baseline characteristics of the participants in the SHARE and HILDA surveys are presented in Table 1. The HILDA population were slightly more male, slightly more educated, had a slightly lower rate of diabetes, slightly higher rates of depression, and a lower baseline mean level of frailty phenotype. The biggest difference between the populations were the rates for Polypharmacy. The Australian population had considerably higher rates of participants reporting that they were taking 5 or more drugs a week over the last 12 months.

**Table 1:** Characteristics of survey participants in SHARE and HILDA datasets.

Characteristics (over 65 years)	SHARE wave 4 (N = 26,530)	HILDA wave 13 (N = 2,701)
Mean age (std dev)	73.93 (73.85, 74.01)	73.49 (73.23, 73.75)
Proportion female	55.71% (55.11, 56.30)	43.47% (41.60, 45.34)
Edu 1	48.83% (48.23, 49.43)	45.17% (43.30, 47.05)
Edu 2 finished school	34.66% (34.09, 3523)	27.18% (25.53, 28.89)
Edu 3 Finished university	16.51% (16.07, 16.96)	27.66% (26.00, 29.38)
Diabetes	16.05% (15.61, 16.49)	11.70% (10.49,12.91)
Depression	7.47% (7.16, 7.79)	11.85% (10.63,13.07)
Stroke	6.03% (5.74, 6.31)	7.29% (6.31,8.27)
Polypharmacy (5 or more drugs)	9.08% (8.74, 9.43)	26.66% (24.99, 28.33)
**Frailty Phenotype Criteria–baseline**		
Weight (appetite)	10.90%	8.15%
Exhaustion	41.03%	26.21%
Physical Activity	25.69%	21.66%
Walking Speed	25.49%	22.62%
Grip Strength	16.46%	17.59%
**FPH 4 category, n = 3,439**		
0	38.62% (38.03, 39.20)	54.31% (52.43, 56.19)
1	28.25% (27.71, 28.79)	19.25% (7.81, 20.78)
2	16.08% (15.64, 16.52)	10.29% (9.20, 11.50)
3	17.06% (16.61, 17.52)	16.14% (14.80, 17.58)
**Average Baseline Frailty**	**1.12 (1.10, 1.13)**	**0.88 (0.84, 0.93)**
**Baseline Events**		
Hosp Admission	18.68%	12.88%
Hip Fracture	3.30%	2.96%*
Falls	7.11%	7.52%*
Disability	16.53%	20.21%
Delirium	1.77%	1.99%*

Imputed values *

Table 2 shows frailty prevalence and mean frailty by age cohorts at baseline across SHARE and HILDA datasets. Table 2 also reports results from an Australian study of frailty as a comparator [33]. In general, frailty rates were lower in the HILDA data than in SHARE or Thompson. While there are around the same proportions of people classified as frail with a frailty phenotype of 3 or more in all three data sets, the HILDA data contains more non-frail and fewer people with 1 or 2 frailty phenotypes and mean frailty is lower in the HILDA population than in the SHARE population at all age groups.

**Table 2:** Frailty prevalence and mean frailty by age categories at baseline across datasets.

	SHARE Observed w4, n = 26,530			Mean Frailty, SHARE, w4	HILDA Observed w13, n = 2,701			Mean Frailty, HILDA, w13	Thompson, 2018, n = 8,804		
Frailty	0	1 & 2	3+		0	1 & 2	3+		0	1 & 2	3+
**Age in years**											
65–69	50.8%	41%	8.2%	0.77 (0.75, 0.80)	63.5%	26.2%	10.3%	0.65 (0.59, 0.72)	58%	38%	5%
70–74	42.4%	45.3%	12.3%	0.97 (0.95, 1.00)	58.3%	28.3%	13.4%	0.78 (0.70, 0.86)	52%	41%	7%
75–79	33.1%	47.4%	19.5%	1.25 (1.22, 1.28)	52.8%	29.8%	17.4%	0.92 (0.82, 1.02)	32%	50%	18%
80–84	24.3%	46.4%	29.3%	1.55 (1.51, 1.59)	37.1%	37.4%	25.5%	1.29 (1.16, 1.42)	24%	50%	26%
85 +	17.9%	43.3%	38.8%	1.81 (1.75, 1.86)	34.1%	36.6%	29.3%	1.52 (1.36, 1.69)			

Source: Thompson et al., Frailty prevalence in Australia: Findings from four pooled Australian cohort studies, Australasian Journal of Ageing, 2018

### Sub-models

The area under the receiver operating characteristics curve (ROC), also known as c-statistics [54], provides an aggregate measure of classification performance across classification thresholds.

The area under the ROCs for each sub-model were:

- Falls model 0.726
- Hospital admission model 0.641
- Hip Fracture model 0.779
- Disability model 0.826
- Delirium model 0.752
- Death model 0.795
- Polypharmacy model 0.820
- Stroke model 0.664

### Internal validation

Tables 3 and 4 show the model predictions of events and updated attributes at 24 months and at 48 months for the wave 4 SHARE population compared to the observed values for that population in wave 5 and wave 6 for males and females respectively. There was overlap in the 95% confidence intervals for the predicted and observed means for all events and updates, except stroke in males aged 65–75 and hospital admissions and mean frailty for females aged over 75. There was overlap in the 99% confidence intervals for these events, but the predicted mean did not lie within 10% of the observed mean.

**Table 3:** Comparisons of model predicted event rates at 24 months and at 48 months with observed rates for the wave 4 males participants in wave 5 and wave 6.

	Observed prevalence			Predicted prevalence		Convergence Criteria	
Statistic	Proportion (95% CI)	(99% CI)	± 10%	Proportion (95% CI)	(99% CI)	Overlapping 95%/99% CIs	Within 10%
Males 65–75 years at 24 months							
Death at 24m	0.033 (0.029, 0.038)	(0.028, 0.039)	(0.030, 0.037)	0.035 (0.031, 0.039)	(0.030, 0.041)	yes/yes	yes
Delirium at 24m	0.032 (0.027, 0.037)	(0.026, 0.039)	(0.029, 0.035)	0.033 (0.029, 0.037)	(0.028, 0.039)	yes/yes	yes
Disability at 24m	0.106 (0.098, 0.115)	(0.095, 0.117)	(0.095, 0.117)	0.112 (0.105, 0.120)	(0.103, 0.122)	yes/yes	yes
Falls at 24m	0.056 (0.050, 0.062)	(0.048, 0.064)	(0.050, 0.061)	0.061 (0.056, 0.067)	(0.054, 0.069)	yes/yes	yes
Hip Fracture at 24m	0.015 (0.012, 0.019)	(0.011, 0.020)	(0.014, 0.017)	0.014 (0.011, 0.017)	(0.010, 0.018)	yes/yes	yes
Hospital Admission at 24m	0.186 (0.175, 0.197)	(0.172, 0.200)	(0.167, 0.204)	0.203 (0.194, 0.212)	(0.191, 0.215)	yes/yes	yes
Polypharmacy at 24m	0.057 (0.051, 0.064)	(0.049, 0.066)	(0.052, 0.063)	0.060 (0.055, 0.066)	(0.053, 0.068)	yes/yes	yes
Stroke at 24m	0.070 (0.064, 0.078)	(0.062, 0.080)	(0.063, 0.077)	0.085 (0.079, 0.092)	(0.077, 0.094)	no/yes	no
Frailty phenotype (mean) at 24m	0.77 (0.74, 0.80)	(0.74, 0.81)	(0.69, 0.85)	0.81 (0.79, 0.83)	(0.78, 0.84)	yes/yes	yes
Males 65–75 years at 48 months							
Death at 48m	0.062 (0.057, 0.068)	(0.055, 0.070)	(0.056, 0.068)	0.073 (0.068, 0.080)	(0.066, 0.082)	yes/yes	no
Delirium at 48m	0.036 (0.031, 0.042)	(0.030, 0.044)	(0.033, 0.040)	0.037 (0.033, 0.042)	(0.031, 0.043)	yes/yes	yes
Disability at 48m	0.117 (0.107, 0.126)	(0.105, 0.130)	(0.105, 0.128)	0.122 (0.114, 0.130)	(0.112, 0.132)	yes/yes	yes
Falls at 48m	0.060 (0.053, 0.067)	(0.051, 0.070)	(0.054, 0.066)	0.075 (0.069, 0.081)	(0.067, 0.083)	no/yes	no
Hip Fracture at 48m	0.012 (0.009, 0.016)	(0.008, 0.017)	(0.011, 0.013)	0.016 (0.014, 0.020)	(0.013, 0.021)	yes/yes	no
Hospital Admission at 48m	0.207 (0.195, 0.219)	(0.191, 0.223)	(0.186, 0.227)	0.215 (0.205, 0.224)	(0.202, 0.228)	yes/yes	yes
Polypharmacy at 48m	0.068 (0.060, 0.075)	(0.058, 0.078)	(0.061, 0.074)	0.069 (0.063, 0.076)	(0.062, 0.078)	yes/yes	yes
Stroke at 48m	0.088 (0.080, 0.097)	(0.077, 0.100)	(0.079, 0.097)	0.110 (0.102, 0.117)	(0.100, 0.120)	no/no	no
Frailty phenotype (mean) at 48m	0.85 (0.82, 0.88)	(0.81, 0.89)	(0.77, 0.94)	0.89 (0.87, 0.92)	(0.86, 0.92)	yes/yes	yes
Males over 75 years at 24 months							
Death at 24m	0.099 (0.090, 0.109)	(0.088, 0.112)	(0.090, 0.109)	0.114 (0.104, 0.124)	(0.102, 0.127)	yes/yes	no
Delirium at 24m	0.036 (0.029, 0.044)	(0.027, 0.047)	(0.033, 0.040)	0.042 (0.036, 0.049)	(0.034, 0.052)	yes/yes	no
Disability at 24m	0.228 (0.211, 0.245)	(0.206, 0.250)	(0.205, 0.250)	0.232 (0.218, 0.246)	(0.214, 0.251)	yes/yes	yes
Falls at 24m	0.124 (0.111, 0.137)	(0.107, 0.142)	(0.111, 0.136)	0.132 (0.121, 0.143)	(0.118, 0.147)	yes/yes	yes
Hip Fracture at 24m	0.027 (0.021, 0.034)	(0.020, 0.037)	(0.025, 0.030)	0.032 (0.027, 0.039)	(0.025, 0.041)	yes/yes	no
Hospital Admission at 24m	0.238 (0.222, 0.256)	(0.217, 0.261)	(0.215, 0.262)	0.254 (0.240, 0.269)	(0.236, 0.274)	yes/yes	yes
Polypharmacy at 24m	0.086 (0.075, 0.098)	(0.072, 0.101)	(0.077, 0.095)	0.089 (0.080, 0.099)	(0.077, 0.102)	yes/yes	yes
Stroke at 24m	0.117 (0.104, 0.130)	(0.101, 0.134)	(0.105, 0.129)	0.122 (0.111, 0.133)	(0.108, 0.137)	yes/yes	yes
Frailty phenotype (mean) at 24m	1.41 (1.37, 1.46)	(1.35, 1.47)	(1.27, 1.55)	1.45 (1.41, 1.49)	(1.40, 1.50)	yes/yes	yes
Males over 75 years at 48 months							
Death at 48m	0.191 (0.179, 0.204)	(0.176, 0.208)	(0.172, 0.211)	0.236 (0.223, 0.249)	(0.219, 0.253)	no/no	no
Delirium at 48m	0.038 (0.030, 0.048)	(0.028, 0.051)	(0.034, 0.042)	0.041 (0.034, 0.049)	(0.032, 0.051)	yes/yes	yes
Disability at 48m	0.233 (0.214, 0.253)	(0.208, 0.259)	(0.210, 0.256)	0.271 (0.255, 0.287)	(0.250, 0.293)	no/yes	no
Falls at 48m	0.127 (0.112, 0.143)	(0.108, 0.148)	(0.114, 0.139)	0.158 (0.145, 0.171)	(0.141, 0.176)	no/yes	no
Hip Fracture at 48m	0.027 (0.020, 0.035)	(0.018, 0.038)	(0.024, 0.029)	0.036 (0.029, 0.043)	(0.027, 0.045)	yes/yes	no
Hospital Admission at 48m	0.234 (0.216, 0.254)	(0.210, 0.261)	(0.211, 0.258)	0.296 (0.280, 0.313)	(0.275, 0.319)	no/no	no
Polypharmacy at 48m	0.077 (0.065, 0.090)	(0.062, 0.094)	(0.069, 0.085)	0.091 (0.081, 0.102)	(0.078, 0.105)	yes/yes	no
Stroke at 48m	0.120 (0.106, 0.135)	(0.101, 0.140)	(0.108, 0.132)	0.159 (0.146, 0.173)	(0.142, 0.177)	no/no	no
Frailty phenotype (mean) at 48m	1.50 (1.45, 1.55)	(1.43, 1.57)	(1.35, 1.65)	1.57 (1.53, 1.61)	(1.52, 1.63)	yes/yes	yes

CI = confidence intervals

**Table 4:** Comparisons of model predicted event rates at 24 months and at 48 months with observed rates for the wave 4 female participants in wave 5 and wave 6.

	Observed prevalence			Predicted prevalence		Convergence Criteria	
Statistic	Proportion (95% CI)	(99% CI)	± 10%	Proportion (95% CI)	(99% CI)	Overlapping 95%/99% CIs	Within 10%
Females 65–75 years at 24 months							
Death at 24m	0.016 (0.013, 0.019)	(0.013, 0.020)	(0.014, 0.018)	0.020 (0.017, 0.023)	(0.016, 0.024)	yes/yes	no
Delirium at 24m	0.067 (0.061, 0.074)	(0.059, 0.076)	(0.060, 0.074)	0.070 (0.065, 0.076)	(0.063, 0.077)	yes/yes	yes
Disability at 24m	0.125 (0.117, 0.134)	(0.115, 0.136)	(0.113, 0.138)	0.130 (0.123, 0.137)	(0.121, 0.140)	yes/yes	yes
Falls at 24m	0.108 (0.100, 0.116)	(0.098, 0.118)	(0.097, 0.119)	0.106 (0.100, 0.113)	(0.098, 0.115)	yes/yes	yes
Hip Fracture at 24m	0.018 (0.015, 0.022)	(0.014, 0.023)	(0.016, 0.020)	0.018 (0.015, 0.021)	(0.015, 0.022)	yes/yes	yes
Hospital Admission at 24m	0.172 (0.163, 0.181)	(0.160, 0.184)	(0.155, 0.189)	0.188 (0.180, 0.197)	(0.178, 0.199)	yes/yes	yes
Polypharmacy at 24m	0.097 (0.090, 0.105)	(0.088, 0.107)	(0.087, 0.107)	0.101 (0.095, 0.108)	(0.093, 0.110)	yes/yes	yes
Stroke at 24m	0.057 (0.052, 0.063)	(0.050, 0.065)	(0.052, 0.063)	0.065 (0.060, 0.070)	(0.059, 0.072)	yes/yes	no
Frailty phenotype (mean) at 24m	1.04 (1.01, 1.07)	(1.01, 1.07)	(0.94, 1.14)	1.09 (1.06, 1.11)	(1.06, 1.12)	yes/yes	yes
Females 65–75 years at 48 months							
Death at 48m	0.035 (0.031, 0.039)	(0.030, 0.040)	(0.031, 0.038)	0.046 (0.042, 0.051)	(0.041, 0.052)	no/no	no
Delirium at 48m	0.083 (0.076, 0.090)	(0.074, 0.093)	(0.075, 0.091)	0.072 (0.067, 0.078)	(0.065, 0.079)	yes/yes	no
Disability at 48m	0.142 (0.134, 0.152)	(0.131, 0.155)	(0.128, 0.157)	0.155 (0.148, 0.163)	(0.145, 0.165)	yes/yes	yes
Falls at 48m	0.121 (0.113, 0.130)	(0.110, 0.132)	(0.109, 0.133)	0.126 (0.119, 0.133)	(0.117, 0.135)	yes/yes	yes
Hip Fracture at 48m	0.020 (0.016, 0.023)	(0.015, 0.025)	(0.018, 0.021)	0.021 (0.018, 0.024)	(0.017, 0.025)	yes/yes	yes
Hospital Admission at 48m	0.185 (0.175, 0.195)	(0.172, 0.198)	(0.166, 0.203)	0.208 (0.200, 0.217)	(0.197, 0.220)	no/yes	no
Polypharmacy at 48m	0.127 (0.119, 0.136)	(0.116, 0.139)	(0.115, 0.140)	0.106 (0.100, 0.113)	(0.098, 0.115)	no/no	no
Stroke at 48m	0.068 (0.061, 0.075)	(0.060, 0.077)	(0.061, 0.075)	0.094 (0.088, 0.100)	(0.086, 0.102)	no/no	no
Frailty phenotype (mean) at 48m	1.14 (1.11, 1.16)	(1.10, 1.17)	(1.02, 1.25)	1.19 (1.17, 1.22)	(1.16, 1.22)	no/yes	yes
Females over 75 years at 24 months							
Death at 24m	0.066 (0.060, 0.073)	(0.058, 0.075)	(0.059, 0.073)	0.072 (0.065, 0.079)	(0.064, 0.081)	yes/yes	yes
Delirium at 24m	0.074 (0.066, 0.083)	(0.063, 0.086)	(0.067, 0.082)	0.079 (0.071, 0.086)	(0.069, 0.089)	yes/yes	yes
Disability at 24m	0.282 (0.267, 0.297)	(0.262, 0.302)	(0.254, 0.310)	0.296 (0.283, 0.309)	(0.279, 0.313)	yes/yes	yes
Falls at 24m	0.203 (0.190, 0.217)	(0.186, 0.221)	(0.183, 0.223)	0.222 (0.211, 0.234)	(0.207, 0.238)	yes/yes	yes
Hip Fracture at 24m	0.048 (0.041, 0.055)	(0.039, 0.058)	(0.043, 0.052)	0.043 (0.038, 0.049)	(0.036, 0.051)	yes/yes	yes
Hospital Admission at 24m	0.227 (0.213, 0.241)	(0.209, 0.246)	(0.204, 0.250)	0.258 (0.246, 0.270)	(0.242, 0.274)	no/yes	no
Polypharmacy at 24m	0.141 (0.130, 0.153)	(0.126, 0.157)	(0.127, 0.155)	0.142 (0.133, 0.152)	(0.130, 0.155)	yes/yes	yes
Stroke at 24m	0.101 (0.092, 0.112)	(0.089, 0.115)	(0.091, 0.112)	0.109 (0.101, 0.118)	(0.098, 0.121)	yes/yes	yes
Frailty phenotype (mean) at 24m	1.77 (1.73, 1.80)	(1.72, 1.82)	(1.59, 1.94)	1.85 (1.82, 1.88)	(1.81, 1.89)	no/yes	yes
Females over 75 years at 48 months							
Death at 48m	0.135 (0.126, 0.144)	(0.123, 0.147)	(0.121, 0.148)	0.160 (0.150, 0.170)	(0.147, 0.173)	no/no	no
Delirium at 48m	0.084 (0.074, 0.096)	(0.071, 0.099)	(0.076, 0.093)	0.084 (0.076, 0.093)	(0.073, 0.095)	yes/yes	yes
Disability at 48m	0.307 (0.290, 0.325)	(0.285, 0.331)	(0.277, 0.338)	0.346 (0.332, 0.360)	(0.327, 0.365)	no/yes	no
Falls at 48m	0.214 (0.199, 0.230)	(0.194, 0.235)	(0.193, 0.235)	0.254 (0.241, 0.267)	(0.237, 0.271)	no/no	no
Hip Fracture at 48m	0.043 (0.035, 0.051)	(0.033, 0.054)	(0.038, 0.047)	0.050 (0.044, 0.057)	(0.042, 0.059)	yes/yes	no
Hospital Admission at 48m	0.224 (0.208, 0.240)	(0.204, 0.245)	(0.202, 0.246)	0.296 (0.282, 0.310)	(0.278, 0.314)	no/no	no
Polypharmacy at 48m	0.155 (0.141, 0.169)	(0.137, 0.174)	(0.139, 0.170)	0.162 (0.151, 0.173)	(0.148, 0.177)	yes/yes	yes
Stroke at 48m	0.123 (0.111, 0.136)	(0.108, 0.140)	(0.111, 0.136)	0.150 (0.140, 0.161)	(0.136, 0.165)	no/yes	no
Frailty phenotype (mean) at 48m	1.84 (1.80, 1.89)	(1.79, 1.90)	(1.66, 2.03)	1.99 (1.96, 2.02)	(1.95, 2.03)	no/no	yes

CI = confidence intervals

At 48 months, however, the model predictions were variable across groups and events. In males 65–75 years, there was overlap in the 95% confidence intervals between the observed and the predicted mean for all events, except falls and stroke, although there was overlap in the 99% confidence intervals for predicted and observed mean for falls. The predictions for death, falls, hip fracture and stroke in males 65–75 years, were more than 10% above the observed mean.

In females 65–75 years, there was only overlap in the 95% confidence intervals between the observed and the predicted mean for delirium, disability, falls and hip fracture, although there was overlap in the 99% confidence intervals for hospital admission and mean predicted frailty fell. Predictions fell more than 10% above observed values for death, hospital admissions and stroke, but below 10% of the observed mean for delirium and polypharmacy at 48 months in this group.

In males over 75 years, there was only overlap in the 95% confidence intervals between observed and predicted incidence for delirium, hip fracture, polypharmacy and mean frailty, although there was overlap in 99% confidence intervals for disability and falls. All predictions for events and attributes, except for delirium and polypharmacy were higher than 10% above the observed mean.

For females over 75 years, there was only overlap in confidence intervals between observed and predicted incidence for delirium and hip fracture and polypharmacy, although there was overlap between the 99% confidence intervals for disability and stroke. All predictions, except for delirium and polypharmacy exceeded 10% above the observed mean in this group.

### External validation

The external validation results for the model using the HILDA data are presented in Table 5, which shows the calibrated results using 1,271 convergent sets of sampled model input parameters. A table of the calibration multipliers applied to the transition probabilities is provided in the appendix. Estimates for the HILDA population from the calibrated model fall within the 95% confidence interval for the observed mean results at 48 months.

**Table 5:** (Calibrated): Predicted mean event rates at 48 months and actual rates for same wave 13 participants in wave 17 survey, (HILDA).

	Observed prevalence	Mean predicted prevalence*	Observed prevalence	Mean predicted prevalence*
	Proportion (95% CI)	Proportion (95% CI)	Proportion (95% CI)	Proportion (95% CI)
**Event**	**Males under 76 years**		**Females under 76 years**	
Death	0.033 (0.023, 0.046)	0.031 (0.030, 0.031)	0.032 (0.021, 0.048)	0.029 (0.028, 0.029)
Delirium	NA	0.042 (0.042, 0.043)	NA	0.090 (0.090, 0.091)
Disability	0.207 (0.179, 0.236)	0.188 (0.187, 0.188)	0.223 (0.189, 0.259)	0.195 (0.195, 0.196)
Falls	NA	0.069 (0.069, 0.069)	NA	0.116 (0.116, 0.116)
Hip Fracture	NA	0.011 (0.010, 0.011)	NA	0.010 (0.010, 0.010)
Hospital Admission	0.130 (0.107, 0.155)	0.134 (0.134, 0.134)	0.139 (0.112, 0.170)	0.146 (0.146, 0.146)
Polypharmacy	0.242 (0.213, 0.273)	0.233 (0.232, 0.233)	0.271 (0.235, 0.310)	0.269 (0.269, 0.270)
Stroke	0.070 (0.053, 0.089)	0.076 (0.076, 0.076)	0.083 (0.062, 0.109)	0.077 (0.077, 0.077)
Frailty phenotype (mean)	0.70 (0.63, 0.77)	0.67 (0.66, 0.67)	0.85 (0.76, 0.94)	0.90 (0.90, 0.90)
	**Males over 75 years**		**Females over 75 years**	
Death	0.133 (0.105, 0.166)	0.136 (0.136, 0.137)	0.140 (0.108, 0.176)	0.147 (0.147, 0.148)
Delirium	NA	0.039 (0.039, 0.039)	NA	0.074 (0.074, 0.074)
Disability	0.232 (0.186, 0.282)	0.254 (0.253, 0.254)	0.269 (0.212, 0.332)	0.280 (0.280, 0.281)
Falls	NA	0.127 (0.127, 0.127)	NA	0.240 (0.240, 0.241)
Hip Fracture	NA	0.033 (0.033, 0.033)	NA	0.043 (0.042, 0.043)
Hospital Admission	0.203 (0.159, 0.252)	0.176 (0.176, 0.177)	0.193 (0.143, 0.251)	0.219 (0.218, 0.219)
Polypharmacy	0.315 (0.264, 0.370)	0.304 (0.303, 0.305)	0.417 (0.352, 0.485)	0.443 (0.442, 0.444)
Stroke	0.119 (0.085, 0.160)	0.102 (0.101, 0.102)	0.126 (0.085, 0.176)	0.125 (0.125, 0.125)
Frailty phenotype (mean)	1.15 (1.02, 1.28)	1.09 (1.09, 1.09)	1.61 (1.44, 1.77)	1.52 (1.52, 1.52)

* based on 1,271 convergent samples

CI = confidence intervals

Table 6 shows the predicted prevalence of each frailty phenotype category and the predicted mean frailty phenotype by age cohort compared to their observed values for the SHARE and the HILDA data. The model predictions were close to observed values for the prevalence of each frailty phenotype category and for mean frailty at 48 months in the SHARE data. The calibrated model predictions were close to observed values for the prevalence of each frailty phenotype category and for mean frailty at 48 months in the HILDA data, except for mean frailty value predicted for males in the age cohort 65–69 years. This was due to the small number of available observations for this age cohort as 65 years is the lowest age of entry for participants in the model, which may be creating the unexpectedly low numbers of males with zero frailty phenotypes in this age category in the observed data.

**Table 6:** Frailty prevalence by age category, predicted and observed values.

	Observed Frailty at 48 months SHARE wave 6				Predicted Frailty at 48 months SHARE wave 6				Observed Frailty at 48 months HILDA wave 17				Predicted Frailty at 48 months HILDA wave 17 (Calibrated)			
Frailty Phenotype	0	1 & 2	3	Mean (CI)	0	1 & 2	3	Mean (CI)	0	1 & 2	3+	Mean (CI)	0	1 & 2	3+	Mean (CI)
**Age Group**	**Males**															
65–69	56.4%	38.1%	5.5%	0.66 (0.63, 0.69)	55.6%	37.7%	6.7%	0.69 (0.62, 0.75)	55.3%	32.1%	12.6%	0.80 (0.59, 1.00)	70.8%	23.0%	6.2%	0.48 (0.32, 0.64)
70–74	50.8%	40.5%	8.7%	0.83 (0.80, 0.87)	49.5%	41.4%	9.1%	0.82 (0.78, 0.85)	62.7%	27.4%	9.9%	0.64 (0.55, 0.74)	58.0%	30.4%	7.6%	0.65 (0.57, 0.73)
75–79	41.8%	44.8%	13.4%	1.10 (1.05, 1.14)	40.5%	44.2%	15.3%	1.05 (1.01, 1.09)	61.3%	26.4%	12.3%	0.74 (0.62, 0.86)	51.9%	39.8%	8.3%	0.77 (0.67, 0.87)
80–84	29.2%	50.3%	20.5%	1.36 (1.30, 1.42)	25.9%	47.4%	26.7%	1.45 (1.40, 1.51)	45.9%	37.0%	17.1%	1.02 (0.85, 1.20)	45.0%	43.2%	11.8%	0.92 (0.78, 1.06)
85 +	18.6%	45.6%	35.9%	1.56 (1.47, 1.65)	20.2%	41.1%	**38.7%**	1.75 (1.68, 1.82)	35.5%	40.4%	24.1%	1.30 (1.11, 1.50)	34.0%	45.8%	20.2%	1.20 (1.05, 1.36)
	**Females**															
65–69	48.6%	41.5%	9.9%	0.87 (0.84, 0.90)	48.4%	42.3%	9.3%	0.83 (0.77, 0.90)	49.4%	37.9%	12.7%	0.89 (0.65, 1.12)	51.4%	38.1%	10.5%	0.81 (0.61, 1.01)
70–74	40.1%	47.3%	12.6%	1.09 (1.06, 1.12)	38.9%	45.8%	15.3%	1.07 (1.04, 1.11)	58.2%	27.3%	14.5%	0.78 (0.66, 0.90)	53.3%	35.6%	11.1%	0.77 (0.67, 0.87)
75–79	30.0%	48.4%	21.6%	1.37 (1.33, 1.41)	26.4%	48.6%	25.0%	1.44 (1.40, 1.48)	48.1%	36.2%	15.7%	0.97 (0.81, 1.13)	34.0%	44.0%	22.0%	1.21 (1.06, 1.36)
80–84	19.7%	47.9%	32.4%	1.69 (1.64, 1.74)	16.5%	45.1%	38.4%	1.81 (1.76, 1.85)	31.1%	45.3%	23.6%	1.37 (1.15, 1.59)	26.0%	39.5%	34.5%	1.62 (1.44, 1.80)
85 +	11.3%	40.3%	48.4%	1.96 (1.89, 2.03)	10.5%	32.2%	57.3%	2.21 (2.16, 2.26)	23.1%	31.6%	45.3%	1.82 (1.59, 2.05)	19.8%	35.1%	45.1%	1.83 (1.64, 2.01)

CI = confidence intervals

## Discussion

This paper reports on the development, validation and calibration of an individual-based state transition model for the prediction of frailty and seven frailty-related events for a cohort of older Australians. Using a model structure previously validated by experts [25], the parameterisation of our simulation model was informed by the experience of a large heterogenous European population (SHARE) with external validation and calibration to a large Australian cohort (HILDA). A strength of our model is that it can estimate outcomes for highly heterogenous patient cohorts using a recognised measure of frailty. Our use of a four factor Frailty phenotype score also makes the model sensitive to transitions between frailty states to better capture intervention effects. The individual-based state transition model is an improvement on the previous Markov model [24] as it can more accurately capture the changing interdependencies between events and individual characteristics over time, which better represents the progression of frailty and its outcomes.

Internal validation showed the model predicted frailty-related events by age and gender at 24 months in the SHARE population with reasonable accuracy but was less accurate at predicting events at 48 months. The model required considerable calibration to accurately predict outcomes and characteristics by age and gender at 48 months for a different population in the external validation, which may be partly explained by differences in the data sources. The HILDA population was less frail at baseline and more highly educated than the SHARE population.

The two data sources used in the model construction share the difficulties associated with self-report, particularly in capturing events over time, with both SHARE and HILDA typically recording binary indicators of characteristics and events to indicate occurrence over a range of specified time frames. These differences are also likely exacerbated by differences in the data collected, particularly in the variables underpinning the construction of the frailty phenotype. The variable which showed the biggest difference in the two groups was the measure for exhaustion. In the SHARE data, the question informing this value involved a comparison of current and previous experience, e.g., “have you had any energy to do the things you used to do?”, whereas for the HILDA data this measure was reliant on a much more general question regarding current levels of energy without reference to previous levels of energy. Other differences include the measure of grip strength, which is an objective measure in the SHARE data, whereas in the HILDA data it is a self-report measure. Conversely the measure of weight loss is based on BMI is the HILDA data, whereas the SHARE data measures this phenotype with self-reported changes in appetite.

Slight differences also occur in the variables used to define characteristics and events in the model. The measure of stroke in the HILDA data includes other serious circulatory conditions, unlike the SHARE data which records stroke specifically. Difference in polypharmacy were even more marked with the HILDA recording the number of prescription medicines taken on a regular basis, compared to SHARE reporting the number of drugs taken in a week for a defined set of conditions.

Another explanation for the model’s poor performance over 48 months duration may be the use of logistic regression models, which assume time-constant monthly prediction of events, which means misrepresentations in the models cumulates the effects over 48 months. The estimation of transition probabilities for events in the model was complicated by the lack of specific time of events. The multinomial logistic model used to predict changes in frailty represented changes in frailty states across all levels of phenotype, for which low numbers of observed transitions between some frailty levels may have reduced the accuracy of the model. While other methods were attempted, such as zero-inflated beta models, these were not found to produce better prediction and made more restrictive assumptions regarding pathways through models. The lack of more specific time to event data and the limited available number of waves at the time of model construction meant that we were unable to fit reliable risk equations using survival curves approaches which would have avoided the need for the assumption of time constancy in the transition probabilities. However, the constructed model is sufficiently flexible to allow for updates to the underlying sub models that underpin the individual-based state-transition model. The application of the regression models over monthly cycles in the simulation model cumulates the effects of any inaccuracies in the models.

With an aging population, Frailty prevalence is likely to increase with detrimental effects on both quality of life for affected people and health service costs. Published results of frailty interventions tend to only consider outcomes during trial duration, but there are likely to be longer-term costs and effects that should be estimated when evaluating frailty interventions. The calibrated model accurately predicts frailty and frailty-related events and attributes by age and gender over 48 months in a community-dwelling Australian population aged 65 and over, with a range of underlying characteristics and event histories. The next steps include the assignment costs and outcomes (e.g., utility values) to the predicted events to enable the model to be used for the economic evaluation of frailty interventions. A longer term goal will be to update the model as more longitudinal data becomes available, such that the evaluation tool will capture important longer term effects of interventions designed to delay or prevent the onset of frailty.

## Supporting information

S1 Table SHARE/ HILDA variable table: Characteristics. (DOCX)

S2 Table SHARE/ HILDA: Event variable table. (DOCX)

S3 Table SHARE/ HILDA frailty phenotype variables. (DOCX)

S4 Table Table of coefficients for logistic models. (DOCX)

S5 Table Multipliers applied in the calibrated model. (DOCX)
