# Supplementary material for: Development and validation of an individual-based state-transition model for the prediction of frailty and frailty-related events
Source: PLoS One. 2023 Aug 24;18(8):e0290567. doi: 10.1371/journal.pone.0290567 (PMC10449188; doi:10.1371/journal.pone.0290567)
Supplement: S1 Table — (DOCX) [file pone.0290567.s001.docx]

**S1 Table. SHARE/ HILDA Variable Table: Characteristics**

|  | **SHARE** | **HILDA** |
| --- | --- | --- |
| Age | Age of respondent at interview | Age at interview, hgage1 |
| Gender | Male/ Female | Male/Female, hgsex1 |
| Education | Level of education | Level of education, edhigh1 |
| Diabetes | Doctor told you: you had diabetes or high blood sugar | Diagnosed with serious illness – (type 2 diabetes), mhedi2=1. |
| Depression | Depression (using drug) | Diagnosed with serious illness – (depression or anxiety), hedep=1. |
| Stroke | **PH006(d4):** Has a doctor ever told you that you had any of the conditions on this card? Please tell me the number or numbers of the conditions.  (Only for stroke)  **No = 0; Yes =1**  **PH009(d4)** AGE WHEN CONDITION STARTED  (Only for stroke) | **heany/heoc:** “Ever been told by a nurse or doctor that you have any of these conditions: Diagnosed with serious illness – any other serious circulatory condition (e.g., stroke or hardening of the arteries)  Please only include those conditions that have lasted or are likely to last six months or more.  **stroke=0 if heany>0**  **stroke=1 if heoc==1** |
| Polypharmacy | **PH011:** Do you currently take drugs at least once a week for problems mentioned on this card?  **(Less than 5 drugs =0; 5 or more drugs = 1)** | **hepmlyr/hepmnum:** “Altogether, how many different prescription medications do you take on a regular basis?”  **Polypharmacy=0 if hepmlyr>0**  **Polypharmacy=1 if hepmnum>=5** |
