# Supplementary material for: Development and validation of an individual-based state-transition model for the prediction of frailty and frailty-related events
Source: PLoS One. 2023 Aug 24;18(8):e0290567. doi: 10.1371/journal.pone.0290567 (PMC10449188; doi:10.1371/journal.pone.0290567)
Supplement: S2 Table — (DOCX) [file pone.0290567.s002.docx]

**S2 Table.** **SHARE/ HILDA: Event Variable Table**

| **Event** | **SHARE** | **HILDA** |
| --- | --- | --- |
| Disability | **PH061:** (Based on ADL and IADL  ADL: based on ph049_1 to ph049_14 which describe number of limitations with activities of daily living  ADL2: reclassifies ADL into 2 categories: 0 no limitations and 1 one or more.  Using ADL2 for disability | **helth/hecrpa:** “Do you have any long-term health condition, impairment or disability (such as these) which restricts you in your everyday activities, and has lasted or is likely to last 6 months or more?”  Which conditions – any condition that restricts physical activity or physical work (e.g., back problems, migraines)  **disability=0 if helth>=0**  **disability=1 if hecrpa==1** |
| Falls | **PH010_d7 (W1, 2 and 4) and PH089_d7 (W5 and 6)?**  For the past six months at least, have you been bothered by any of the health conditions on this card? Please tell me the number or numbers  **No = 0; Yes =1**  (Bothered by falling down) | NA |
| Delirium | (W1, 2 and 4): **MH021**_ EVER ADMITTED TO MENTAL HOSPITAL OR PSYCHIATRIC WARD Have you been admitted in mental hospital or psychiatric ward?  **1. Yes**  **5. No**  **Ph006-18 (w5 and W6)** | NA |
| Hip Fracture | **PH006_14:** Has a doctor ever told you that you had any of the conditions on this card? Please tell me the number or numbers of the conditions.  (Only for hip fracture)  **No=0**  **Yes=1** | NA |
| Hospital Admission | **HC012:** During the last twelve months, have you been in a hospital overnight?  **1. Yes**  **5. No**  IF HC012 = 1 (Yes)  **HC013**: How often (how many times) have you been a patient in a hospital overnight during the last twelve months? 1-10 | **phonpat/hehan:** During the last 12 months, have you ever been a patient in a hospital overnight?  **hosp=0 if phonpat>0**  **hosp=1 if hehan==1** |
