# Supplementary material for: Development and validation of an individual-based state-transition model for the prediction of frailty and frailty-related events
Source: PLoS One. 2023 Aug 24;18(8):e0290567. doi: 10.1371/journal.pone.0290567 (PMC10449188; doi:10.1371/journal.pone.0290567)
Supplement: S3 Table — (DOCX) [file pone.0290567.s003.docx]

**S3 Table. SHARE/ HILDA Frailty Phenotype Variables**

| **SHARE wave 4** | **HILDA wave 13** |
| --- | --- |
| **Weight Loss** |  |
| mh011 appetite 1. “Diminution in desire for food” (self-reported)  mh012 eating more or less than normal 1. ‘Eating less than usual”  Pheno_crit_1 = 1 if mh011=1 OR mh012=1 | BMI self-report  Pheno_crit_1 = 1 if BMI<21 |
| **Exhaustion** |  |
| mh013=1 “Have you had little energy to do the things you used to do?”  (yes=1)  Pheno_crit_2 = 1 if mh013=1 | SF36: gh9e: Vitality: have lots of energy  Responses: 1. All of the time, 2. Most of the time, 3. A good bit of the time, 4. Some of the time, 5. A little of the time, 6. None of the time  Pheno_crit_2 = 1 if gh9e = 5 or 6 |
| **Physical Activity** |  |
| br016  “how often do you engage in activities that require a moderate level of energy such as gardening, cleaning the car, or going for a walk”  Responses: 3. 1-3 times a month, 4. hardly ever, never  Pheno_crit_3 = 1 if br016=3 or br016= 4 | gh3b SF-36 moderate activities  gh3h Hlth limits walking 500m   1. Limited a lot, 2. Limited a little, 3. Not limited at all   Pheno_crit_3 = 1 if: gh3b = 1 OR gh3h = 1 |
| **Walking speed** |  |
| ph048d1=1 Difficulties walking 100m (directly measured) (1. Yes)  ph048d5=1 Difficulties climbing a flight of stairs (1. Yes)  Pheno_crit_4 = 1 if ph048d1=1 OR ph048d5=1 | gh3e Health limits climbing stairs, one flight (more: gh3d)  gh3i Health limits walking 100m  Responses: 1. Limited a lot, 2. Limited a little, 3. Not limited at all  Pheno_crit_4 = 1 if: gh3i = 1 OR gh3e = 1 OR (gh3i = 2 AND gh3e = 2) |
| **Grip strength** |  |
| gs001 “Willingness to do grip strength test”  (3. unable to take measurement)  gs002 == (2. unable to use RH, 3. unable to use LH)  max_gs maximum of gs006 – gs009  BMI & gender criteria | gh3c SF-36 lifting or carrying groceries  Responses: 1. limited a lot, 2. Limited a little, 3. Not limited at all)  hedgt Long term health condition. Difficulty gripping things (1.yes)  Pheno_crit_5 = 1 if: hedgt = 1 OR gh3c = 1 |
