# Supplementary material for: Development and validation of an individual-based state-transition model for the prediction of frailty and frailty-related events
Source: PLoS One. 2023 Aug 24;18(8):e0290567. doi: 10.1371/journal.pone.0290567 (PMC10449188; doi:10.1371/journal.pone.0290567)
Supplement: S4 Table — (DOCX) [file pone.0290567.s004.docx]

**S4 Table. Table of coefficients for Logistic Models**

|  | Falls | Hospital Adm | Hip Fracture | Disability | Delirium | Death | Polypharmacy | Stroke |
| --- | --- | --- | --- | --- | --- | --- | --- | --- |
| Frailty ph 1 | 0.393 (0.067) | 0.240 (0.049) | 0.325 (0.144) | 0.461 (0.067) | 0.808 (0.098) | 0.622 (0.154) | 0.681 (0.084) | 0.374 (0.121) |
| Frailty ph 2 | 0.582 (0.077) | 0.392 (0.061) | 0.607 (0.158) | 1.028 (0.073) | 1.021 (0.113) | 1.030 (0.159) | 1.054 (0.093) | 0.585 (0.139) |
| Frailty ph 3 | 0.808 (0.083) | 0.559 (0.069) | 0.841 (0.175) | 1.613 (0.076) | 1.343 (0.119) | 1.487 (0.158) | 1.359 (0.101) | 0.856 (0.145) |
| Age | 0.046 (0.004) | 0.018 (0.003) | 0.043 (0.008) | 0.053 (0.004) | -0.013 (0.006) | 0.070 (0.006) | 0.303 (0.096) | 0.414 (0.160) |
| Age sq |  |  |  |  |  |  | -0.002 (0.001) | -0.003 (0.001) |
| Gender | 0.496 (0.054) | -0.148 (0.040) |  |  | 0.525 (0.078) | -0.753 (0.088) | 0.353 (0.062) | -0.226 (0.094) |
| Polypharmacy |  | 0.345 (0.070) |  | 0.213 (0.083) | 0.349 (0.112) |  | 1.829 (0.079) | 0.344 (0.148) |
| Stroke | 0.247 (0.100) | 0.243 (0.081) |  | 0.645 (0.088) |  |  | 0.485 (0.107) |  |
| Educ 2 |  | 0.172 (0.044) |  | -0.028 (0.054) |  | 0.018 (0.096) |  |  |
| Educ 3 |  | 0.191 (0.055) |  | -0.142 (0.070) |  | -0.322 (0.149) |  |  |
| Diabetes | 0.299 (0.065) | 0.184 (0.053) |  | 0.250 (0.065) | -0.203 (0.100) |  | 0.872 (0.068) | 0.519 (0.110) |
| Depression | 0.319 (0.087) |  |  | 0.434 (0.090) | 1.607 (0.097) | 0.496 (0.119) | 0.386 (0.099) |  |
| Hospital Adm |  | 0.706 (0.046) | 0.278 (0.120) |  |  | 0.619 (0.090) | 0.321 (0.07) | 0.271 (0.108) |
| Hip Fracture |  |  | 2.645 (0.127) | 0.458 (0.116) |  |  |  |  |
| Falls | 1.007 (0.079) | 0.245 (0.076) |  | 0.455 (0.086) |  |  |  | 0.336 (0.159) |
| Delirium |  |  |  |  | 0.667 (0.180) |  |  |  |
| Disability | 0.443 (0.068) | 0.177 (0.059) | 0.302 (0.135) | 1.737 (0.058) |  | 0.433 (0.101) | 0.200 (0.080) |  |
| Constant | -6.938 (0.306) | -3.050 (0.246) | -7.681 (0.556) | -6.746 (0.288) | -3.744 (0.423) | -9.121 (0.501) | -15.98 (3.627) | -20.298 (6.067) |
| ROC | 0.726 | 0.641 | 0.779 | 0.826 | 0.752 | 0.795 | 0.820 | 0.664 |
