# Supplementary material for: Development and validation of an individual-based state-transition model for the prediction of frailty and frailty-related events
Source: PLoS One. 2023 Aug 24;18(8):e0290567. doi: 10.1371/journal.pone.0290567 (PMC10449188; doi:10.1371/journal.pone.0290567)
Supplement: S5 Table — (DOCX) [file pone.0290567.s005.docx]

**S5 Table.** **Multipliers applied in the Calibrated Model**

| **Event** | **Males under 76 years** | **Females under 76 years** | **Males over 75 years** | **Females over 75 years** |
| --- | --- | --- | --- | --- |
|  |  |  |  |  |
| Death | .6 | .7 | .7 | 1.1 |
| Disability | 1.55 | 1.3 | 1 | .95 |
| Frailty: |  |  |  |  |
| 0 to 0 | 2.1 | 2.1 | 2.1 | 2.1 |
| 0 to 1 | .75 | .75 | .75 | 1.0 |
| 0 to 2 | .4 | .4 | .4 | .5 |
| 0 to 3 | .4 | .4 | .4 | .4 |
| 1 to 0 | 1.15 | 1.15 | 1.15 | 1.15 |
| 1 to 1 | 1 | 1 | 1 | 1 |
| 1 to 2 | .66 | .66 | .66 | .9 |
| 1 to 3 | .33 | .33 | .33 | .45 |
| 2 to 0 | 1.15 | 1.15 | 1.15 | 1.15 |
| 2 to 1 | 1 | 1 | 1 | 1 |
| 2 to 2 | 1 | 1 | 1 | 1 |
| 2 to 3 | .66 | .66 | .66 | .9 |
| 3 to 0 | 1 | 1 | 1 | 1 |
| 3 to 1 | 1 | 1 | 1 | 1 |
| 3 to 2 | 1 | 1 | 1 | 1 |
| 3 to 3 | 1 | 1 | 1 | 1 |
| Hospital | .55 | .675 | .75 | .75 |
| Polypharmacy | 2.9 | 2.1 | 2.6 | 2.3 |
| Stroke | .4 | .4 | .4 | .4 |
